# Daily Snacking Occasions, Snack Size, and Snack Energy Density as Predictors of Diet Quality among US Children Aged 2 to 5 Years

**DOI:** 10.3390/nu11071440

**Published:** 2019-06-26

**Authors:** Alexandria Kachurak, Regan L. Bailey, Adam Davey, Lauren Dabritz, Jennifer Orlet Fisher

**Affiliations:** 1 Center for Obesity Research and Education, College of Public Health, Temple University, 3223 N. Broad Street, Suite 175, Philadelphia, PA 19140, USA; 2 Department of Nutrition Science, College of Health and Human Services, Purdue University, 700 West State Street, West Lafayette, IN 47907, USA; 3 Department of Behavioral Health and Nutrition, University of Delaware, 016 Carpenter Sports Building, Newark, DE 19716, USA; 4 Center for Obesity Research and Education, College of Public Health, Temple University, 3223 N. Broad Street, Suite 175, Philadelphia, PA 19140, USA; 5 Department of Social and Behavioral Sciences, Center for Obesity Research and Education, College of Public Health, Temple University, 3223 N. Broad Street, Suite 175, Philadelphia, PA 19140, USA

**Keywords:** snacking, preschool, children, diet quality, healthy eating index, mean adequacy ratio, shortfall nutrients, added sugar, sodium, saturated fat

## Abstract

Whether snacks help young children meet nutritional needs or merely contribute to excessive intakes is debated. This research evaluated associations of snacking with dietary quality among US preschoolers (two to five years, *n* = 4217) in the 2005–2016 National Health Examination Survey (NHANES). Snacking occasions, size, and energy density (ED) were estimated from two 24-hr dietary recalls. Diet quality indices included the 2015 Healthy Eating Index (HEI-2015, 0–100), the mean adequacy ratio (MAR, 0–100) for five shortfall nutrients (vitamin D, calcium, fiber, potassium, and iron), and the mean % of recommended limits for added sugars, saturated fat, and sodium. Linear regressions included snacking parameters, demographics, and dietary reporting accuracy. Children had a mean HEI-2015 of 53.0, a MAR of 67.7, and intake of 121.4% of nutrients to limit. Daily snacking occasions were positively associated with HEI-2015 scores, whereas mean snack size and ED were negatively associated with HEI-2015 and MAR scores (all *p* < 0.05). Snack ED was positively associated with daily intakes of added sugar, saturated fat, and sodium (*p* < 0.001). These nationally representative findings reveal that more frequent, smaller, and less energy-dense snacks are associated with higher diet quality among US preschoolers.

## 1. Introduction

Snacking, broadly defined as eating in between meals, is not normative for children in some countries [1], but is nearly universal among US children. By 12 months of age, nearly 95% of US children consume one or more snacks on a given day [2]. By preschool, the contribution of snacks to children’s daily energy intake is similar to that of any other single meal. In 2016, snacking provided 26–27% of daily energy among US children aged two to five years [3]. Given its central contribution to daily energy intake, whether snacking plays an “essential” or “excessive” role in children’s diets is an important nutritional consideration, particularly for young children for whom snacks are believed to have most nutritional relevance for growth and development [4].

The extent to which young children need snacks is debated, with two nearly opposite and prevailing views. The view of snacks as “essential” holds that young children have small stomachs and need snacks to meet nutrient requirements for growth. This view is reflected in the American Academy of Pediatrics (AAP) Healthy Active Living for Families obesity prevention-oriented guidance that includes provisions of two daily snacks for preschool aged children [5]. Further, the US Department of Agriculture’s MyPlate guidance for healthy eating includes snacks ideas in its recommendations for young children (www.choosemyplate.gov). Among US children aged two to five years in 2015–2016, snacking occasions provided roughly one-quarter or more of children’s daily intake of a number of key shortfall nutrients [3].

Alternatively, the view of snacks as “excessive” holds that snacking may contribute to obesity by providing “extra” calories to young children’s diets. This position is supported by secular increases in the frequency of and energy consumed from snacking occasions among US children since the 1970′s [6], along with observations that the top foods consumed by US children at snacking occasions are desserts, sweetened beverages, and salty snacks [7,8,9].

To date, there has been surprisingly little empirical evidence of snacking influences on young children’s dietary quality. A handful of studies of older children and adolescents, reveal associations of snacking frequency and snack energy density (ED) with intake of specific food groups and nutrients, as well as overall diet quality [10,11,12,13,14]. However, research on younger children has been mostly descriptive. For instance, using NHANES 2005–2012 data (*n* = 3429), Shriver and colleagues determined that the sweets food group (e.g., cookies and pastries) and sugar-sweetened beverages were the top sources of energy and added sugar consumed as snacks by US children aged two to five years [8]. While snacks made significant contributions (>25%) to children’s daily intakes of vitamin C, potassium, and fiber, snacking occasions also provided close to 40% of children’s daily intakes of added sugars and 27% of children’s intakes of saturated fat. Thus, evidence to date is limited and mixed, highlighting the need for research to better understand how snacking contributes to the overall dietary quality of young children.

This study aimed to evaluate associations of snacking among US preschool-aged children with overall diet quality and daily intake of nutrients of public health concern using NHANES 2005–2016 data. Specifically, we used the 2015 Healthy Eating Index (HEI-2015) [15] to understand the extent to which snacking may influence the extent to which children’s dietary intake is aligned with current 2015–2020 US Dietary Guidelines for Americans (DGA) [16]. In addition, we characterized children’s daily intake of five “essential” shortfall nutrients (e.g., iron, vitamin D, calcium, fiber, potassium) as well as three “excessive” nutrients to limit (e.g., added sugar, saturated fat, and sodium) as identified in the 2015 DGA [17]. This research also advances previous work that has typically focused on single dimensions of snacking (e.g., frequency) by evaluating unique contributions of multiple snacking parameters including the number of daily snacking occasions, the mean snack size (mean energy consumed per occasion), and the mean ED of foods/beverages per snacking occasion (mean energy per gram per occasion). To our knowledge, this research is the first nationally representative study to comprehensively characterize snacking contributions to diet quality in young children.

## 2. Materials and Methods

### 2.1. Design and Participants

Participants were US children aged two to five seen in one of six consecutive cycles of NHANES from 2005–2016. NHANES is a continuous cross-sectional nationally representative study of the nutritional and health status of the civilian non-institutionalized US population [17,18]. NHANES uses a complex, multistage, probability sampling design with county as the primary sampling unit within which clusters of households and then participants are selected. NHANES includes an in-home interview, clinical measurements at a Mobile Exam Center, and a follow-up telephone call. Response rates across the 2005–2016 cycles ranged from 61.3% to 80.5% (median = 75.5%). Written consent for children’s participation was provided by the parent/legal guardian as approved by the National Center for Health Statistics Research Ethics Review Board.

Children between the ages of 24.0 and 71.9 months were eligible for the study. Exclusion criteria were diabetes (*n* = 4) and having less than one day of dietary data (*n* = 700). Dietary data which included occasions labeled as “infant feeding” were also excluded (*n* = 59), as many involved solely milk feedings (e.g., human milk, formula). The final analytic sample included 4217 cases with dietary data and design weights.

### 2.2. Measures

Demographics. Data on age, sex, and race/ethnicity were collected for the participating child and head of the household during the in-home interview. Data on marital status, level of education, and income were collected about the head of the household. The poverty income ratio (PIR) was estimated to express household income relative to the poverty level, with adjustments for family size, year, and state [19].

Dietary intake. Children’s dietary intake was assessed as part of the What We Eat in America (WWEIA) [20] survey component of NHANES using two 24-h dietary recalls. The first recall occurred in-person in the mobile exam center, and a second recall occurred by telephone. The validated automated multiple pass methodology was used including a quick list recall, forgotten foods, eating time and frequency, detail cycle, and final probe [21]. A proxy respondent (e.g., parent or caregiver from the home) completed dietary recalls on behalf of the participating child. Data were processed using the US Department of Agriculture Food and Nutrient Database for Dietary Studies (Versions 3.0, 4.1, 5.0, 2010–2011, 2013–2014, and 2015–2016). Mean two-day values are presented, and dietary weights were employed to represent both days of data, adjusted for non-response and day of the week [22]. Nutrient intakes from supplements were not included given the focus of this analysis on eating behavior and on nutrient contributions from foods and beverages alone.

Snacking Parameters. Eating occasions were participant-defined from a pre-determined list. Consistent with previous studies, all food and beverages reported at the same clock time were considered a single eating occasion [23,24]. Consistent with our previous research [25], daily snacking occasions were defined as the number of daily occasions labeled as “snack” as well as other food/beverage occasions between meals as given by “beverage”, and “extended consumption” labels (and Spanish equivalents). Occasions at which trivial energy (<5 kcal) was consumed (e.g., water) were excluded. Mean snack size, calculated as the mean energy (kcal) consumed per snacking occasion, was used to describe the caloric content of snacking occasions. Mean snack ED (kcal/g) was calculated as mean energy per gram of food/beverage consumed per snacking occasion [26] to provide a general proxy of the quality of snacks consumed. In cases where no snacks were reported (*n* = 102, 2.1%), mean snack size and mean ED were set to missing such that values represent characteristics of snacks consumed.

### 2.3. Dietary Outcomes

Overall dietary quality. The HEI-2015 assesses the alignment of dietary intake with recommendations of the 2015 DGA involving the consumption of a variety of food groups, nutrient density, and improving food and beverage choices within calorie needs [15]. HEI-2015 consists of 13 component scores of which nine reflect recommendations around adequacy (i.e., total fruit, whole fruit, total vegetables, greens and beans, whole grains, dairy, total protein foods, seafood and plant proteins, and fatty acids) and four reflect recommendations for moderation (i.e., refined grains, sodium, added sugar, and saturated fats). Component scores are summed such that possible scores range from 0 to 100, with higher scores indicating greater alignment with recommendations of the 2015 DGA [16]. The survey years included in this analysis span the HEI-2010 and HEI-2015; we chose to utilize the most recent HEI.

Shortfall nutrients. Nutrient adequacy ratios (NAR) were calculated for five shortfall nutrients (potassium, fiber, calcium, vitamin D, and iron) identified in the 2015 DGA [16]. The NAR for each nutrient was calculated as an individual’s daily intake relative to the nutritional goals for that nutrient [27], using the Recommended Dietary Allowance values for calcium, vitamin D and iron, and Adequate Intake values for potassium and fiber [28,29]. Values were truncated at 100, indicating that intake was 100% of the recommendation. The mean adequacy ratio (MAR) was calculated as the mean NAR for the five shortfall nutrients, with higher scores indicating greater daily intake of the shortfall nutrients relative to the nutritional recommendations for those nutrients.

Nutrients to limit. Daily intake expressed as mean % of recommended limits for added sugar (<10% of daily energy) and saturated fat (<10% of daily energy) per recommendations in the 2015 DGA [16] and sodium (2–3 years: <1500 mg, 4–5 years: <1900 mg) using the tolerable upper intake level (UL) specified in the Dietary Reference Intakes [28]. Scores indicate the percentage of the recommended limits consumed, such that a score of 110 would indicate a mean intake of 110% of the recommended.

Dietary reporting accuracy. Dietary reporting accuracy was estimated to adjust for reporting bias using the ratio of reported energy intake to estimated energy requirements (EI:EER) [23]. Estimated energy requirements were calculated using Dietary Reference Intake equations based on age, sex, weight, and physical activity level [30]. A “low active” level of physical activity (≥1.4 to <1.6) was assumed for all children based on accelerometer data from NHANES 2003–2006 [31]. Following the methods of Murakami [23], the EI:EER ratio was used as a covariate as this approach has been shown to yield similar results to excluding implausible cases without biasing sample selection [23,32].

Weight status. Weight status was calculated for descriptive purposes. Child weight and standing height were measured by trained professionals at the mobile exam center [33]. Weight status was classified following Centers for Disease Control (CDC) age-specific reference data [34] and recommendations [35]. Normal weight was defined as a BMI-for-age <85th percentile. Overweight was calculated as BMI-for-age 85th to <95th percentiles, and obesity was defined as a BMI-for-age > 95th percentile.

### 2.4. Statistical Analyses

All analyses were performed in Stata/SE (Version 15.0, College Station, TX, USA). Descriptive statistics and measures of variability were generated for all variables. Data are shown as mean (SE) with 95% CI unless otherwise indicated. Linear regression with backward elimination (*p* > 0.10) was used to identify demographic and design covariates. Survey cycle, EI:EER, child gender, age, and race as well as head of the household age, marital status, education, and PIR were retained. Total daily energy intake was also included as a covariate in models predicting children’s intake of shortfall nutrients and nutrients to limit. Multiple linear regression evaluated the associations of snacking parameters (daily snacking occasions, mean snack size, and mean snack ED) with the three diet quality outcomes (HEI-2015, MAR for shortfall nutrients, and % of recommended limits consumed for added sugar, saturated fat, and sodium). Each model included all three snacking parameters in addition to the covariates detailed above. Statistical significance was inferred by *p*-values < 0.05. MAR and % of recommended limits consumed outcomes used square root transformation to approximate normal distributions.

## 3. Results

Demographics. A little more than half of children were non-Hispanic white (53%), with 14–16% non-Hispanic Black or Mexican American (Table 1). Additionally, approximately half of the children were male (51%) and three-fourths were at a normal weight (77%). The mean head of the household age was 36 years. The mean PIR indicated a mean household income of 230% of the federal poverty level. Over half (~60%) of adult head of households reported education beyond high school and 82% were partnered.

Snacking characteristics. On average, children consumed snacks at 2.6 (0.02 SE; 95% CI, 2.5–2.6) occasions daily, with mean snack size of 166.3 kcal per occasion (2.5 SE; 95% CI, 161.3–171.3), and a mean snack ED of 2.5 kcal/g per occasion (0.01 SE; 95% CI, 2.4–2.5). The number of daily snack occasions was weakly negatively correlated with mean snack size (r = −0.13, *p* < 0.0001) and mean snack ED (r = −0.16, *p* < 0.0001), whereas mean snack size and mean snack ED were weakly positively correlated (r = 0.13, *p* < 0.0001).

Dietary quality. Table 2 presents descriptive data on indicators of children’s dietary quality. Children’s overall diet quality was relatively low, as indicated by the mean HEI-2015 score of 53.0 (0.3). The mean MAR representing children’s daily intake of five shortfall nutrients was 67.7 (0.3). Alternatively, children’s daily intakes of sodium, added sugar, and saturated fats exceeded recommended limits, where mean intake across all three nutrients was 121.4 (0.6)% of the recommendations.

Associations of snacking parameters with diet quality. As shown in Table 3, dietary quality among US preschoolers was associated with multiple parameters of snacking. Daily snacking occasions were positively associated with children’s HEI-2015 scores, reflecting overall diet quality (Table 3, *p* < 0.05). Significant positive associations were seen with 5 of the 13 HEI-2015 component scores (Table S1). Alternatively, mean snack size (Table 3, *p* < 0.05) and mean snack ED (Table 3, *p* < 0.01) were negatively associated with children’s HEI-2015 scores. Negative associations of mean snack size and mean ED were seen with 3 of 13 and 6 of 13 HEI-2015 component scores, respectively (Table S1).

Mean snack size was negatively associated with children’s MAR scores, reflecting daily intake of five shortfall nutrients (Table 3, *p* < 0.001). Negative associations of mean snack size were seen with two of five individual NARs (Table S2). Similarly, mean snack ED was negatively associated with MAR scores (Table 3, *p* < 0.001). Negative associations of mean snack ED were seen with three of five individual NARs (Table S2). Daily snacking occasions were unassociated with MAR scores.

Finally, mean snack ED was positively associated with the % of recommended limits consumed for added sugar, saturated fat, and sodium (Table 3, *p* < 0.001). Neither daily snack occasions nor mean snack size were associated with the % of recommended limits. Associations of snacking parameters were inconsistent when looking at individual nutrients to limit (Table S3). For example, the number of daily occasions, snack size, and snack ED were positively associated with children’s consumption of added sugar relative to limits but unassociated with saturated fat intakes, and inconsistently associated with sodium intakes (Table S3).

## 4. Discussion

Understanding the role that snacking plays in the diet quality of young children is critical given that roughly one out of every four calories consumed by US preschool-aged children are currently eaten outside of meals [3]. A recent review of snacking recommendations from 49 countries and 7 global/regional organizations worldwide, revealed that the most frequent rationale given in support of snacking was the potential value for energy and/or nutrient intakes [4]. This possibility has remained largely speculative where young children are concerned given that empirical inquiry to date has been limited and primarily descriptive [3]. The findings of this research provide new evidence that parameters of snacking are associated with overall diet quality, children’s daily intake of shortfall nutrients, and consumption of “excessive” nutrients for which limits are recommended among US children aged two to five years. Using NHANES 2005–2016 data, children’s consumption of more frequent, smaller, and less energy-dense snacks was positively associated with HEI-2015 scores for overall diet quality. Similarly, the consumption of smaller and less energy-dense snacks was associated with higher MAR scores, reflecting greater intakes of five shortfall nutrients. Alternatively, children’s consumption of more energy-dense snacks was positively associated with daily intakes of added sugar, fat, and sodium. Collectively, these nationally representative findings reveal that more frequent, smaller, and less energy-dense snacks are associated with higher diet quality among US preschoolers.

Most of the research on snacking and diet quality has focused on the frequency of snacking. To our knowledge, the present analysis is the first to consider whether the size of snacks consumed by young children may influence diet quality. This analysis revealed that children’s consumption of larger snacks was associated with lower overall diet quality and lower intake of shortfall nutrients. We also observed that children’s consumption of higher ED snacks was associated with lower overall quality, lower intake of shortfall nutrients, and higher intake of added sugar, saturated fat, and salt. The findings regarding snack ED are consistent with two analyses of British population-based data from children 4 to 18 years in the 1997 National Diet and Nutrition Study. In one analysis data, the ED of both snacks and meals consumed by children was inversely associated with total daily intakes of vegetables, fruits, and fiber as well as with overall dietary quality. Associations were stronger, however, for the ED of meals than for snacks [11]. A separate nutrient profiling analysis revealed that the nutritional quality of snacks was lower than that of meals and associated with a poorer nutritional profile of children’s intakes of particular food groups (e.g., dairy, fruits, vegetables, and nuts) and nutrients (e.g., starch, sodium), but was unassociated with overall dietary quality [10]. Collectively, the evidence to date suggests that the quality of snacks consumed by young children has important implications for dimensions of diet quality. Whereas lower ED snacks may positively contribute to young children’s intake of nutrients of public health concern and overall diet quality, higher energy-dense snacks may contribute to excessive intakes of added sugar, saturated fat, and sodium.

When accounting for mean snack size and mean snack ED, the results revealed that more frequent snacking was associated with higher HEI-2015 scores for overall diet quality. This finding is counter to results reported in several population-based studies of snacking among older children and adolescents. British data from the 1997 National Diet and Nutrition Survey revealed that greater snacking frequency (occasions providing <15% of daily energy intake) among children 4 to 18 years was associated with higher intakes of soft drinks, confectionery and total sugar, lower intakes of cereals, protein, fiber, and lower overall diet quality [12]. Another study of British adolescents revealed that overall diet quality decreased with increased frequency of daily snacking, but only when low energy-containing snacks (<50 kcal) were excluded from the analysis [14]. Similarly, among US adolescents, more frequent snacking was negatively associated with fruit and vegetable intake and positively associated with sugar-sweetened beverage intake [13]. One reason our findings may have differed from the previous work is that our model simultaneously estimated parameters for snacking frequency, size, and ED. However, we conducted a sensitivity analysis that removed mean snack size and ED from the model and found that the positive association of snack frequency with HEI-2015 scores persisted. Differences between the present and past findings could also suggest that snacks play a more positive role in the diet quality of younger versus older children. Additional research is required, however, to evaluate this possibility. It is also important to note the present and previous studies not only differed in terms of age of children studied but also the cultural context and the years during which the data were collected.

While the findings highlight the potentially positive contributions of smaller, less energy-dense snacks to young children’s diet quality, this notion appears somewhat aspirational at present. Indeed, the top sources of snack energy among US children 2 to 18 years are currently desserts, sugar-sweetened beverages, and salty snacks [7,8,9]. There is behavioral evidence to suggest that young children are inclined to select these types of highly palatable snacks over healthier options when given the choice. An experimental study that varied the types of snacks offered to 5–10-year-old children over two-week periods, found that fruit was seldom selected when offered with sugar-sweetened or salty snacks [36]. Finally, there is also evidence that highly palatable snacks are frequently used to manage young children’s behavior (e.g., as a reward, to keep the child quiet) [37,38] and the use of snacks for non-nutritive purposes has been associated with low adherence to dietary principles emphasized in obesity prevention [39]. Some data indicate that snacks consumed outside of the home, particularly in the afternoon, may represent important contextual targets for addressing snack quality and size. Data from the 2008 Feeding Infants and Toddlers study revealed that US toddlers and preschoolers consume more calories from snacks eaten away from home than at home [40]. Among children 4–13 years participating in 2009–2012 NHANES, afternoon snacking occasions were the most energy-dense and least nutrient-dense [9]. Research is needed to better understand the contextual factors and approaches to feeding young children that can be used to improve the quality of snacks offered to children.

Several strengths and limitations qualify the findings. First, the cross-sectional nature of the NHANES precludes inferences regarding causality. These findings, however, provide direction for more rigorous longitudinal and experimental designs. Second, the inclusion of multiple snacking parameters advances previous research on snacking, which has primarily focused on frequency. While snack ED is a potentially important proxy of the quality of snacks in the context of obesity prevention [41], however, it does not adequately capture the nutritional quality of snacks. Future work should include nutrient profiling to more fully characterize the healthfulness of snacks consumed by young children [42]. Third, we used indicators of diet quality that incorporated both food groups and nutrients to characterize potential contributions to dietary adequacy and excess. While the HEI-2015 is a well-accepted measure of global diet quality, the use of the total score may obscure potentially valuable information about individual components and patterns of dietary quality. Associations of snacking parameters with individual HEI-2015 components are provided in Supplementary Table S1. Fourth, the definition of snacking employed in this analysis was occasion-based using labels categorically assigned by participants which is inherently subjective. The lack of consensus on definitions of snacking has been identified as a barrier to understanding associations with dietary and weight outcomes among children [43,44]. Finally, this analysis relies on self-reported dietary data that have well-known sources of measurement error [45]. While the 24HR recall represents the least-biased estimation method for energy intakes [46] and the automated multi-pass method has been validated in adults [21,47,48], less is known about reporting error among children.

In conclusion, these nationally representative data provide evidence that snacking is associated with the dietary quality of US preschoolers. The findings provide important qualification to the notion that snacks help young children meet nutrient needs. In particular, the results suggest that more frequent, smaller, and less energy-dense snacks may have benefits for diet quality among US preschoolers. These findings provide population representative “signals” for working towards the identification of optimal snacking patterns that maximize the likelihood of nutritional adequacy and minimize the risks of excessive consumption and obesity. Longitudinal and experimental studies are ultimately needed to evaluate potential causal influences of snacking parameters on dietary quality in young children.

## Figures and Tables

**Table 1:** Demographic characteristics (*n* = 4217 ^1^).

	*n*	%
**Child**		
Race		
Non-Hispanic white	1267	53.3
Mexican American	1087	16.0
Non-Hispanic Black	976	14.4
Other Race	461	8.3
Other Hispanic	426	8.1
Gender		
Male	2143	50.6
Female	2074	49.4
Weight status ^2^		
Normal weight	3013	76.6
Overweight	525	12.7
Obese	469	10.7
**Head of Household**		
Age, mean (se)	35.6 (0.3)	
Family ratio of poverty to income, mean (se) ^3^	2.3 (0.1)	
Education		
High school or less	2056	40.6
More than high school	2034	59.5
Marital status		
Partnered	3064	81.9
Not partnered	1004	18.1

^1^ Sample sizes for individual variables reflect missing data. ^2^ Normal weight = BMI-for-age <85th percentile. Overweight = BMI-for-age 85th to <95th percentile. Obese = BMI-for-age ≥ 95th percentile [34,35]. ^3^ Calculated by dividing household income by the Health and Human Services’ poverty guideline, specific to family size, year, and state.

**Table 2:** Dietary quality and intake of selected nutrients among U.S. children aged two to five years (*n* = 4217).

	**Recommendation ^1^**		**Daily Intake**	
	1–3 year	4–8 year	Mean	SE
**HEI-2015 ^2^**	-	-	53.0	0.3
**Shortfall nutrients**				
Fiber (g/d)	19	25	11.6	0.1
Potassium (mg/d)	3000	3800	1991.4	17.0
Calcium (mg/d)	700	1000	955.9	10.2
Iron (mg/d)	7	10	11.5	0.1
Vitamin D (IU)	600	600	247.2	3.7
**MAR ^3^**	-	-	67.7	0.3
**Nutrients to limit**	**Recommendation ^4^**		**Daily Intake**	
	1–3 year	4–8 year	Mean	SE
Sodium (mg/d)	<1500	<1900	2261.3	19.7
Added sugar (% daily energy)	<10%		12.8	0.1
Saturated fat (% daily energy)	<10%		11.7	0.1
**% of recommended limits consumed ^5^**			121.4	0.6

^1^ Recommended dietary allowance (calcium, iron, Vitamin D) or adequate intakes (fiber, potassium) [28,29]. ^2^ Possible scores range from 0 to 100, with higher scores indicating greater alignment with 2015 DGA [16]. ^3^ Mean adequacy ratio (MAR) [27] of reported to recommended intakes [28,29] for five shortfall nutrients per 2015 DGA [16]. ^4^ Tolerable upper intake level (sodium) [28] or limits specified in the 2015 DGA (added sugar, saturated fat) [16]. ^5^ Mean daily intakes of added sugar, saturated fat, and sodium, expressed as the % of recommended limits [16].

**Table 3:** Association between snacking parameters and diet quality among U.S. children aged two to five years (*n* = 3679 ^1^).

	Model 1			Model 2			Model 3		
	HEI-2015 ^2^			Shortfall Nutrients ^3^			Nutrients to Limit ^4^		
	b	SE	*t*	b	SE	*t*	b	SE	*t*
**Daily snacking occasions ^5^**	0.52	0.23	2.24 *	0.003	0.001	1.71	0.92	0.49	1.89
**Mean snack size ^6^**	−0.01	0.003	−2.03 *	−0.0001	0.00003	−3.54 ***	0.02	0.01	1.91
**Mean snack energy density ^7^**	−1.04	0.38	−2.75 **	−0.02	0.003	−4.97 ***	2.21	0.66	3.34 ***

b = unstandardized beta weight, SE = standard error for the unstandardized beta, t = *t* test statistic. * *p* < 0.05, ** *p* < 0.01, *** *p* < 0.001. ^1^ Reflects sample sizes for the following covariates: All models adjusted for dietary weights, survey cycle year, child gender, child age, child race, HH age, HH marital status, HH level of education, ratio of income to poverty, and ratio of reported energy intake to estimated energy requirements. Models 2 and 3 also adjusted for total daily energy intake. ^2^ HEI-2015 scores range from 0 to 100 [15], with higher scores indicating greater alignment of dietary intake with 2015 DGA [16]. ^3^ Ratio [27] of daily intakes to age-specific recommended dietary allowances (calcium, iron, and vitamin D) or adequate intake (fiber, potassium) [28,29] for shortfall nutrients per 2015 DGA [16]. ^4^ Mean daily intakes of added sugar, saturated fat, and sodium, expressed as the % of recommended limits per 2015 DGA [16]. ^5^ Number of daily snacking occasions as given by “snack”, “beverage”, or “extended consumption” labels, excluding trivial energy (<5 kcal) occasions (e.g., water). ^6^ Mean energy (kcal) consumed per snacking occasion. ^7^ Mean energy (kcal) per gram of foods/beverages consumed per snacking occasion.

## Supplementary Materials

Supplementary materials can be found at https://www.mdpi.com/2072-6643/11/7/1440/s1, Table S1. Associations of snacking parameters with HEI-2015 component scores among US children aged two to five years (*n* = 3679), Table S2. Associations of snacking parameters with nutrient adequacy ratios for five shortfall nutrients among US children aged two to five years (*n* = 3679), Table S3. Associations of snacking parameters with children’s % of recommended limits consumed from added sugar, saturated fat, and sodium among US children aged two to five years (*n* = 3679).
