# Supplementary material for: Daily Snacking Occasions, Snack Size, and Snack Energy Density as Predictors of Diet Quality among US Children Aged 2 to 5 Years
Source: Nutrients. 2019 Jun 26;11(7):1440. doi: 10.3390/nu11071440 (PMC6683020; doi:10.3390/nu11071440)
Supplement: Supplementary file 1 [file nutrients-11-01440-s001.pdf]

Supplemental Table 1. Associations of snacking parameters with Healthy Eating Index-2015 (HEI-2015) component scores among US children aged 2-5 y (n=3,679)[1]

	Maximum Score	HEI-2015 component score	Daily Snacking Occasions			Mean snack size			Mean snack energy density		
		Mean (SE) (95% CI)	b	SE	t	b	SE	t	b	SE	t
**HEI-2015 adequacy components**											
Whole fruit	5	3.54 (0.06) (3.43, 3.65)	0.15	0.04	4.02***	-0.0002	0.0006	-2.34	-0.27	0.05	-4.96***
Total Fruit	5	3.84 (0.04) (3.76, 3.92)	0.15	0.04	3.92***	-0.0005	0.001	-0.84	-0.25	0.05	-5.15***
Total vegetables	5	2.01 (0.04) (1.94, 2.08)	-0.08	0.03	-2.64**	-0.002	0.0005	-4.03***	-0.03	0.04	-0.91
Greens and beans	5	1.24 (0.06) (1.13, 1.35)	-0.004	0.04	-0.09	-0.001	0.0007	-1.91	0.09	0.06	1.47
Whole grains	10	2.93 (0.07)	-0.04	0.05	-0.73	0.0002	0.001	0.13	0.19	0.10	1.81

		2.78, 3.08									
Dairy	10	8.31 (0.06) (8.19, 8.42)	0.14	0.05	2.70**	-0.001	0.0009	-1.29	-0.24	0.11	-2.22*
Total protein foods	5	3.54 (0.03) (3.48, 3.60)	-0.18	0.03	-6.28***	-0.0005	0.0004	-1.25	-0.04	0.04	-0.95
Seafood and plant proteins	5	2.08 (0.06) (1.95, 2.20)	-0.07	0.04	-1.51	0.0000004	0.0007	0.00	0.11	0.07	1.55
Fatty acids	10	1.42 (0.08) (1.26, 1.57)	-0.20	0.07	-2.83	-0.0006	0.001	-0.60	0.15	0.10	1.52
**HEI-2015 moderation components**											
Refined grains	10	6.10 (0.08) (5.95, 6.25)	0.42	0.06	7.55***	-0.00006	0.001	-0.05	-0.36	0.10	-3.76***
Sodium	10	5.65 (0.07) (5.51, 5.79)	0.05	0.05	8.55***	0.004	0.001	3.94***	-0.034	0.09	-0.39
Added sugar	10	6.94 (0.06) (6.82, 7.07)	-0.28	0.07	-4.15***	-0.004	0.001	-3.07**	-0.17	0.08	-2.08*
Saturated fats	10	5.42 (0.09) (5.23, 5.61)	-0.01	0.07	-0.17	-0.000001	0.0009	-0.00***	-0.20	0.09	-2.26*

b=unstandardized beta weight, SE=standard error for the unstandardized beta, t=*t* test statistic; *p<0.05; **p<0.01; ***p<0.001

[1]Reflects sample sizes for the following covariates: dietary weights, survey cycle year, child gender, child age, child race, HH age, HH marital status, HH level of education, ratio of income to poverty, and ratio of reported energy intake to estimated energy requirements

[2]HEI-2015 consists of 13 component scores reflecting adequacy and moderation; maximum scores for each component score are either 5 or 10[15]

[3]Number of daily snacking occasions as given by “snack”, “beverage”, or “extended consumption” labels, excluding trivial energy (<5 kcal) occasions (e.g. water)

[4]Mean energy (kcal) per snacking occasion

[5]Mean energy (kcal) per gram of foods/beverages consumed per snacking occasion

Supplemental Table 2. Associations of snacking parameters with nutrient adequacy ratios for 5 shortfall nutrients among US children aged 2-5 y (n=3,679)[1]

	Nutrient adequacy ratio[2]	Daily snacking occasions[3]			Mean snack size[4]			Mean snack energy density[5]		
	Mean (SE) (95% CI)	b	SE	t	b	SE	t	b	SE	t
**Potassium**	58.76 (0.46) (57.85, 59.68)	1.03	0.27	5.62***	-0.01	0.004	1.19	-3.27	0.41	-8.07***
**Fiber**	52.84 (0.52) (51.81, 53.87)	-1.09	0.41	-1.47**	-0.02	0.01	-2.29***	-0.41	0.56	-1.00
**Calcium**	87.26 (0.41) (86.44, 88.08)	0.57	0.30	3.24	-0.01	0.004	-0.11*	-1.54	0.52	-3.21**
**Vitamin D[6]**	40.77 (0.59) (39.60, 41.94)	2.04	0.50	4.85***	-0.003	0.005	1.09	-4.27	0.60	-7.26***
**Iron**	93.85 (0.27) (93.29, 94.38)	-0.40	0.25	-0.56	-0.005	0.004	0.21	0.64	0.35	1.54

b=unstandardized beta weight, SE=standard error for the unstandardized beta, t= *t* test statistic; *p<0.05; **p<0.01; ***p<0.001

[1]Reflects sample sizes for the following covariates: dietary weights, survey cycle year, child gender, child age, child race, HH age, HH marital status, HH level of education, ratio of income to poverty, ratio of reported energy intake to estimated energy requirements, and mean total daily energy

[2]Ratio of daily nutrient intake to the Recommended Dietary Allowance (calcium, iron, Vitamin D) or Adequate Intake (fiber, potassium) [28,29]

[3]Number of daily snacking occasions as given by "snack", "beverage", or "extended consumption" labels, excluding trivial energy (<5 kcal) occasions (e.g. water)

[4]Mean energy (kcal) per snacking occasion

[5]Mean energy (kcal) per gram of foods/beverages consumed per snacking occasion

[6]n=3,437

Supplemental Table 3. Associations of snacking parameters with children's % of recommended limits consumed from added sugar, saturated fat, and sodium among US children aged 2-5 y (n=3,679)[1]

	Mean % of recommended limits consumed[2]	Daily snack occasions[3]			Mean snack size[4]			Mean snack ED[5]		
	Mean (SE) (95% CI)	b	SE	t	b	SE	t	b	SE	t
**Added sugar**	128.35 (1.49) (125.39, 131.31)	6.66	1.57	4.20***	0.09	0.03	2.77**	4.25	1.98	2.18*
**Saturated fat**	116.70 (0.92) (114.88, 118.53)	0.26	0.74	0.51	0.01	0.01	0.83	1.89	0.97	1.91
**Sodium**	119.02 (1.04) (116.96, 121.08)	-4.04	0.46	-4.16***	-0.04	0.01	-0.23***	0.58	0.88	-0.05

b=unstandardized beta weight, SE=standard error for the unstandardized beta, t= *t* test statistic; *p<0.05, **p<0.01, ***p<0.001

[1]Reflects sample sizes for the following covariates: dietary weights, survey cycle year, child gender, child age, child race, HH age, HH marital status, HH level of education, ratio of income to poverty, ratio of reported energy intake to estimated energy requirements, and mean total daily energy

[2]Mean daily nutrient intakes as the % of recommended limits per the 2015-2020 Dietary Guidelines for Americans[16] for added sugar and saturated fat and the tolerable upper intake level (UL) for sodium[28]

[3]Number of daily snacking occasions as given by “snack”, “beverage”, or “extended consumption” labels, excluding trivial energy (<5 kcal) occasions (e.g. water)

[4]Mean energy (kcal) per snacking occasion

[5]Mean energy (kcal) per gram of foods/beverages consumed per snacking occasion
